# Supplementary material for: Unequal access to opioid agonist treatment and sterile injecting equipment among hospitalized patients with injection drug use-associated infective endocarditis
Source: PLoS One. 2022 Jan 26;17(1):e0263156. doi: 10.1371/journal.pone.0263156 (PMC8791472; doi:10.1371/journal.pone.0263156)
Supplement: S1 File — (DOCX) [file pone.0263156.s001.docx]

**Supplementary materials for the article:**

Unequal access to opioid agonist treatment and sterile injecting equipment among hospitalized patients with injection drug use-associated infective endocarditis

Thomas D. Brothers^1,2*^, Kimiko Mosseler^3^, Susan Kirkland^1,4^, Patti Melanson^5†^, Lisa Barrett^1,6^, Duncan Webster^1,7^

^1^Department of Medicine, Dalhousie University, Halifax, Nova Scotia, Canada

^2^UCL Collaborative Centre for Inclusion Heath, Institute of Epidemiology and Health Care, University College London

^3^Dalhousie Medicine New Brunswick, Dalhousie University, Saint John, New Brunswick, Canada

^4^Department of Community Health & Epidemiology, Dalhousie University, Halifax, Nova Scotia, Canada

^5^Mobile Outreach Street Health (MOSH), Halifax, Nova Scotia, Canada

^6^Division of Infectious Diseases, Nova Scotia Health, Halifax, Nova Scotia, Canada

^7^Division of Infectious Diseases, Saint John Regional Hospital and Dalhousie University, Saint John, New Brunswick, Canada

*Corresponding author:
E-mail: thomas.brothers.20@ucl.ac.uk (TDB)

^†^Deceased

**This material supplements, but does not replace, the peer-reviewed article in PLOS ONE.**

**Supplemental Table S1. International Classification of Diseases, Tenth Revision, Canada (ICD-10-CA) codes with a discharge diagnosis potentially consistent with infective endocarditis.**

| **ICD-10-CA** | **Description** |
| --- | --- |
| I011 | Acute rheumatic endocarditis |
| I020 | Rheumatic chorea with heart involvement |
| I058 | Other mitral valve diseases |
| I059 | Mitral valve disease, unspecified |
| I068 | Other rheumatic aortic valve diseases |
| I069 | Rheumatic aortic valve disease, unspecified |
| I078 | Other tricuspid valve diseases |
| I080 | Disorders of both mitral and aortic valves |
| I081 | Disorders of both mitral and tricuspid valves |
| I082 | Disorders of both aortic and tricuspid valves |
| I083 | Combined disorders of mitral, aortic and tricuspid valves |
| I088 | Other multiple valve diseases |
| I089 | Multiple valve disease, unspecified |
| I091 | Rheumatic diseases of endocardium, valve unspecified |
| I098 | Other specified rheumatic heart diseases |
| I33 | Acute and subacute endocarditis |
| I348 | Other nonrheumatic mitral valve disorders |
| I358 | Other aortic valve disorders |
| I368 | Other nonrheumatic tricuspid valve disorders |
| I378 | Other pulmonary valve disorders |
| I38 | Endocarditis, valve unspecified |
| I391 | Aortic valve disorders in diseases classified elsewhere |
| I393 | Pulmonary valve disorders in diseases classified elsewhere |
| I398 | Endocarditis, valve unspecified, in diseases classified elsewhere |
| I423 | Endomyocardial (eosinophilic) disease |
