# Supplementary material for: Unequal access to opioid agonist treatment and sterile injecting equipment among hospitalized patients with injection drug use-associated infective endocarditis
Source: PLoS One. 2022 Jan 26;17(1):e0263156. doi: 10.1371/journal.pone.0263156 (PMC8791472; doi:10.1371/journal.pone.0263156)
Supplement: Attachment — Submitted filename: IDUIE_response_R1_211129.docx [file pone.0263156.s002.docx]

Tara Gomes, PhD
Academic Editor, *PLOS One*

29-Nov-2021

**Re: Manuscript PONE-D-21-27677**

Dear Dr. Gomes,

Thank you for considering our manuscript and for the opportunity to revise and re-submit. We appreciate the Reviewers’ close reading of the manuscript and their specific and helpful suggestions, and we feel they have improved the quality of the manuscript. We have attempted to address each, and we provide a point-by-point response, below. We have itemized and underlined the changes (line numbers refer to those in the “tracked changes” version of the manuscript, showing “All markup”).

**Reviewer 1 comments:**

Thank you for the opportunity to review this manuscript, which uses a retrospective chart review design to look at several indicators of substance use care in hospital including OAT and harm reduction/sterile supply distribution. Strengths of this manuscript include some interesting findings regarding substance use in hospital as well as leaving against medical advice, and it highlights the need to improve care for people who inject drugs in hospital. My recommendations to strengthen the manuscript are as follows:

**Comment 1.1:** Style: Methods line 92 and further below, the word opioid agonist treatment is spelled out yet the acronym OAT is used above – would continue throughout

**Authors’ response 1.1:** We have made this change throughout, as suggested.

**Comment 1.2:** Introduction: I am more familiar with the term opioid agonist therapy rather than opioid agonist treatment. The former would be my suggestion.

**Authors’ response 1.2:** While we use the terms “opioid agonist treatment” and “opioid agonist therapy” interchangeably in everyday use and clinical practice, we have left OAT as “opioid agonist treatment” in this manuscript because this is the terminology used in the current Canadian opioid use disorder treatment guidelines (Bruneau et al., CMAJ, 2018, https://doi.org/10.1503/cmaj.170958)

**Comment 1.3:** Methods: Can you clarify how you searched the ICD-10 codes? Does each hospital have a database that codes discharge diagnosis?

**Authors’ response 1.3:** Clarification added. Administrative records with ICD-10 codes for relevant hospitalizations were identified by clinical informatics analysts at each institution. Canadian hospitals (except Quebec) follow national standards for discharge abstract coding and reporting to the Canadian Institute for Health Information (CIHI), using ICD-10-CA codes.

- Methods, page 6, lines 191-193:

“Clinical informatics analysts searched administrative records at both institutions to identify hospitalizations for patients with a discharge diagnosis code potentially consistent with IE, via International Classification of Diseases, Tenth Revision (ICD-10) codes (Supplemental Table S1).

**Comment 1.4:** Overall, more detail is needed on how the chart review was performed. Are these electronic or paper records?

**Authors’ response 1.4:** Details added:

- Methods, page 4, lines 146-150:

“One investigator at each site (TDB and KM) performed structured chart review, extracting information on demographic and clinical factors, substance use and treatment history. This information was extracted from multidisciplinary team progress notes, specialist consultation records, physician orders, medication administration records, and hospital discharge summaries. All documentation in Halifax was available as paper charts scanned to an online system, and in Saint John was combination of physical paper charts and electronic progress notes.”

**Comment 1.5:** The definition of untreated opioid use disorder used is “documented injection opioid use without current use of OAT”. However, opioid use disorder requires a patient to meet certain DSM-V criteria beyond just use of opioids. Anyone using opioids by injection is thereby labelled in this study as having an OUD. Would suggest clarifying the limitation that your definition is not able to differentiate between those with a DSM-V diagnosis of OUD vs. opioid use alone.

**Authors’ response 1.5:** Clarification added to limitations section in Discussion:

- Discussion, Limitations, page 16, lines 454-457:

“Second, we used self-reported injection opioid use as our operational definition of opioid use disorder (and therefore to identify patients eligible for OAT) since many patients did not have other documentation of diagnostic criteria for substance use disorders.”

**Comment 1.6:** In line 125-126, what is meant by “current use of OAT”? Can you specify the exact medications you considered to be OAT here (presumably methadone, bup/nx, and SROM based on the lines below) and on what time frame did they have to have a prescription? Was this based on a prescription database or patient self report?

**Authors’ response 1.6:** Clarification provided:

- Methods, page 6, lines 203-207:

“We assessed medical records from IDU-IE hospitalizations to determine the frequency of patients with untreated opioid use disorder, which we defined as documented injection opioid use without an active prescription for OAT (methadone, buprenorphine-naloxone, or slow-release oral morphine) at the time of patients’ presentation to hospital. At both hospitals, patients’ self-report of OAT prescription and dosage is confirmed with their community pharmacy before doses are provided.”

**Comment 1.7:** The authors mention that medical records were assessed for “documentation of hospital-based healthcare providers offering sterile drug injecting equipment”. I think the manuscript would be strengthened with further information around this. Is this something you would expect is typically documented in the chart? What healthcare providers are permitted to distribute this and is there a protocol for documentation? Were all interdisciplinary progress notes reviewed? Again, more details on the charting system and records for these hospitals would be very helpful. Given that the authors found NO documented offers of the provision of sterile injection equipment, it raises the possibility that this is simply not something that is typically documented in a chart even if a conversation is had… (especially since there is a policy for such in Saint John)

**Authors’ response 1.7:** We agree that more information would be helpful for readers, since inpatient needle exchange policies are still so rare (even in Canada). We have added more information about the policy in Saint John that was active during the study period by including a copy of the policy as a new Figure 1 and by adding more detail in the Methods and Discussion. As the policy was explicitly framed as “staff protection initiative” at the time (rather than as a patient-centred harm reduction initiative), it was typical to document activation of the policy in the multidisciplinary progress notes so that the team would be aware of needles in the patient’s room. As described in our response to Reviewer 1’s comment #4 (above), we have tried to make it clearer that all of this multidisciplinary progress note documentation was reviewed. In Halifax, there was no policy related to substance use, and so if hospital-based providers were providing sterile injecting equipment surreptitiously this would still be a failure to incorporate evidence-based best practices into the care plan. Indeed, in Halifax we found documentation of nurses confiscating patients’ injecting equipment that was presumably provided by the local government funded harm reduction outreach program based only a few blocks away from the hospital. We have also clarified this limitation further in the Discussion:

- Methods, page 5, lines 165-166:

“The version of the relevant policy (“Intravenous Needle Exchange Policy”) that was active during the study period is presented in Fig 1.”

- Methods, page 5, lines 171-172:

**“Fig 1. Policy enabling inpatient needle and syringe distribution at Saint John Regional Hospital during the study period**.”

- Discussion, page 16, lines 449-454:

“Our study has some important limitations. First, our data came from retrospective chart review, and it is possible that hospital-based providers offered sterile injecting equipment and did not document this in the medical record. However, in Saint John the needle exchange policy at the time (Fig 1) was framed as “staff protection initiative” and so it was typical to document provision of needles to patients. At either site, if sterile injecting equipment were provided surreptitiously and not documented, this would still represent a failure to incorporate evidence-based best practices into the care plan.”

- Discussion, page 16, lines 440-445:

“The findings of the present study are now informing the development of comprehensive substance use and harm reduction policies at both hospitals. The needle exchange policy in Saint John has since been revised to reframe its focus from a “staff protection initiative” to a component of patient-centered care for people who use drugs. Best practices for hospital inpatient harm reduction policies include distribution of alcohol swabs, tourniquets, filters, cookers, sterile water, and vitamin C (which may not otherwise be on hospital formularies), in addition to needles and syringes [7,11].”

- Discussion, page 15, lines 339-401:

“We identified no documented evidence that patients with IDU-IE were offered or provided sterile injecting equipment by hospital-based health care providers at either hospital, despite a long-standing policy to facilitate this in Saint John.”

**Comment 1.8:** Results: The study is from October 2015 – March 2017. Given that this is now several years old, discussion on how things may have changed in these settings and hospitals since then (or not) would be helpful, particularly given the escalation of the overdose crisis.

**Authors’ response 1.8:** We have made changes to the Discussion to highlight developments since the 2015-2017 study period:

- Discussion, page 14, lines 376-384:

“Despite several years passing since our study period, the removal of Health Canada’s certification requirement to prescribe methadone for OAT, and Canada’s escalating overdose death crisis, the QEII Health Sciences Centre in Halifax still does not have addiction medicine-trained physicians or OAT providers on staff. This is typical of many hospitals in North America. Specialized addiction medicine consultation services can help close this gap, by providing expertise, increase uptake of these interventions, and assist in linking hospitalized patients to outpatient addictions treatment [25,26,32,42–44]. In response to identified gaps in care (informed by the present study), hospital-based resident physicians at the Halifax site partnered with community-based addiction physicians to provide a volunteer (unfunded) addiction medicine consultation service [27,45].

- Discussion, page 16, lines 440-445:

“The findings of the present study are now informing the development of comprehensive substance use and harm reduction policies at both hospitals. The needle exchange policy in Saint John has since been revised to reframe its focus from a “staff protection initiative” to a component of patient-centered care for people who use drugs. Best practices for hospital inpatient harm reduction policies include distribution of alcohol swabs, tourniquets, filters, cookers, sterile water, and vitamin C (which may not otherwise be on hospital formularies), in addition to needles and syringes [7,11].”

**Comment 1.9:** The findings in table 2 of rates of illicit or non-medical substance use in hospital and patient-initiated discharges are interesting; while a small study, this detail is a worthwhile contribution to the limited literature in this area.

**Authors’ response 1.9:** Thank you for encouraging us to highlight this. With this in mind, we created a separate paragraph in the Discussion to consider ongoing substance use in hospital, distinct from the paragraph specifically on facilitating needle exchange programs in hospital:

- Discussion, pages 15-16, lines 421-450:

“Such a setting of mutual mistrust and undertreated pain and opioid withdrawal contributes to high rates of illicit or non-medical substance use in hospital and discharges against medical advice among hospitalized patients who use drugs [22,53,54,57–59]. Hospitals have been conceptualized as high-risk environments for people who use drugs [5,58], as abstinence-based policies and threats of punishment lead to high-risk behaviours like using drugs alone in locked bathrooms, rushing injections and missing veins, and taking a bigger dose than usual to try to make it last longer [57–59]. We found that documentation regarding illicit or non-medical substance use in hospital was common at both hospital sites, despite universal access to OAT in Saint John. This suggests that OAT is necessary but not sufficient to support the safety of hospitalized patients who inject drugs; policies that support pain management and harm reduction for ongoing substance use in hospital are also needed [7,11,50–52]. The findings of the present study are now informing the development of comprehensive substance use and harm reduction policies at both hospitals. The needle exchange policy in Saint John has since been revised to reframe its focus from a “staff protection initiative” to a component of patient-centered care for people who use drugs. Best practices for hospital inpatient harm reduction policies include distribution of alcohol swabs, tourniquets, filters, cookers, sterile water, and vitamin C (which may not otherwise be on hospital formularies), in addition to needles and syringes [7,11].”

**Comment 1.10:** Discussion: In line 220, the authors assert that “for patients with IDU-IE, addiction is the underlying cause of their infection”. Some patients who inject drugs may not meet criteria for an opioid use disorder (or substance use disorder) and the definition used by the authors for “OUD” may not actually meet DSM-V criteria. While I agree it is highly likely that the patients’ injection drug use caused the infection, asserting that the “addiction” is the cause is a loaded statement that cannot be readily proven in this study and would suggest rephrasing.

**Authors’ response 1.10:** Suggested change made in this place and one other place:

- Introduction, page 3, lines 82-83:

“Hospitals differ in the care they offer patients with medical complications of addiction and injection drug use, including IDU-IE [7,25–27]”

- Discussion, page 12, line 298:

“For patients with IDU-IE, unsafe injection drug use is the underlying cause of their infection and addiction treatment should be incorporated into treatment plans for secondary prevention [31,32].”

**Reviewer 2 comments:**

The authors performed a retrospective chart review of patients admitted in two Canadian Maritime hospitals with endocarditis associated with injection drug use. This study is a quality control measure looking at access to opioid agonist therapy and sterile injection equipment for inpatients. The authors demonstrate that opioid agonist treatment is not always offered to eligible patients at both hospitals, and sterile injection equipment is not offered to this cohort despite pre-existing hospital policy. The strengths of this work include availability of detailed demographics of the cohort, as well as details on opioid agonist therapy initiation in hospital, and thorough review of the available literature in the discussion. The work’s weakness is the small number of patients included.

**Comment 2.1:** Page 5, line 116-117, description of participants selection: It would be helpful to provide further description of how cases were verified. How many charts using ICD criteria alone were initially isolated? Is medical record completely electronic such that everything required to verify Duke criteria is available easily, including, for instance, blood culture results and vital signs? Or, was all the information gathered from physician documentation only, such that the authors were not able to directly verify laboratory results?

**Authors’ response 2.1:** In response to this comment and Reviewer 1’s comment #4, we have added further description on the chart review procedures and moved this information to a common location in under “data sources”. We were unsure if we had access to all laboratory results, but fortunately all patients were seen by infectious diseases physicians who summarized the relevant culture results in their consultation note.

- Methods, page 4, lines 146-150:

“One investigator at each site (TDB and KM) performed structured chart review, extracting information on demographic and clinical factors, substance use and treatment history. This information was extracted from multidisciplinary team progress notes, specialist consultation records, physician orders, medication administration records, and hospital discharge summaries. All documentation in Halifax was available as paper charts scanned to an online system, and in Saint John was combination of physical paper charts and electronic progress notes.”

We have also added a paragraph with further information on the cases that were excluded. Our study protocol between the sites did not account for keeping track of the total number of charts reviewed or non-injection drug use-associated endocarditis cases identified, but we do have that information available for the Halifax site only. This prevents us from including a traditional flow diagram (e.g.., number of charts screened, etc.), but seems like important information to include.

- Results, page 10, lines 232-236:

“A further three patients in Halifax and six patients in Saint John were hospitalized with IE during the study period with a documented remote history (>3 months prior) of injection drug use, so were excluded from this study. At the Halifax site only, data was captured on the total number of infective endocarditis hospitalizations; the 21 IDU-IE hospitalizations represent 30% of all 71 infective endocarditis hospitalizations during the study period in Halifax.”

**Comment 2.2:** Page 5, line 120: The authors should explain why patients who died in hospital were excluded from the analysis.

**Authors’ response 2.2:** We added an explanation, and moved this whole statement to the subsection specific to that part of the analysis:

- Methods, page 7, lines 194-196:
- “Patients who died in hospital were excluded from the analysis of access to OAT and sterile injecting equipment, because many remained critically ill and/or sedated throughout their hospital stay and so would have been unlikely to access either intervention.”

**Comment 2.3:** Page 6, line 131: Where would the authors expect provision of sterile drug injecting equipment be documented? It may be helpful to include this detail.

**Authors’ response 2.3:** In response to this comment and to Reviewer 1’s comment #7. We have added more detail on the Saint John hospital needle exchange policy (including a copy of the policy itself) and expectations around documentation. As we describe above, as the policy was explicitly framed as “staff protection initiative” at the time (rather than as a patient-centred harm reduction initiative), it was typical to document activation of the policy in the multidisciplinary progress notes. In Halifax, there was no policy related to substance use, and so if hospital-based providers were providing sterile injecting equipment surreptitiously we would still interpret this as a failure to incorporate evidence-based best practices into the care plan. Indeed, in Halifax we found documentation of nurses confiscating patients’ injecting equipment that was presumably provided by the local government funded harm reduction outreach program based only a few blocks away from the hospital. We have also clarified this limitation further in the Discussion.

- Methods, page 5, lines 165-166:

“The version of the relevant policy (“Intravenous Needle Exchange Policy”) that was active during the study period is presented in Fig 1.”

- Methods, page 5, lines 171-172:

**“Fig 1. Policy enabling inpatient needle and syringe distribution at Saint John Regional Hospital during the study period**.”

- Discussion, page 16, lines 449-454:

“Our study has some important limitations. First, our data came from retrospective chart review, and it is possible that hospital-based providers offered sterile injecting equipment and did not document this in the medical record. However, in Saint John the needle exchange policy at the time (Fig 1) was framed as “staff protection initiative” and so it was typical to document provision of needles to patients. At either site, if sterile injecting equipment were provided surreptitiously and not documented, this would still represent a failure to incorporate evidence-based best practices into the care plan.”

- Discussion, page 16, lines 440-445:

“The findings of the present study are now informing the development of comprehensive substance use and harm reduction policies at both hospitals. The needle exchange policy in Saint John has since been revised to reframe its focus from a “staff protection initiative” to a component of patient-centered care for people who use drugs. Best practices for hospital inpatient harm reduction policies include distribution of alcohol swabs, tourniquets, filters, cookers, sterile water, and vitamin C (which may not otherwise be on hospital formularies), in addition to needles and syringes [7,11].”

- Discussion, page 15, lines 339-401:

“We identified no documented evidence that patients with IDU-IE were offered or provided sterile injecting equipment by hospital-based health care providers at either hospital, despite a long-standing policy to facilitate this in Saint John.”

**Comment 2.4:** Page 12, lines 248-254: The chief reason for difference in provision of OAT that the authors cite here is lack of or presence of a specific provider who routinely prescribes OAT. The authors should comment on other barriers to OAT for inpatients. As a physician working in the Maritimes, does one need to have a special license to prescribe either suboxone or methadone? Is addiction medicine part of the standard curriculum in medical school and residency for family physicians, general internists, or anyone else? Why is it that everyone is able to treat hypertension, for instance, but a much smaller proportion of physicians are able to treat opioid addiction?

**Authors’ response 2.4:** Thank you for raising these important points. We have added further description in the Methods (Setting) section describing the regulatory environment around prescribing methadone or buprenorphine during the study period, and we have added further detail in the Discussion to highlight barriers to more widespread availability of OAT in hospitals (including education):

- Methods, page 5, lines 142-147:

“During the study period, both provinces’ public medication insurance programs requested that patients trial methadone before they would pay for buprenorphine; this has since been changed as both medications are considered first line OAT options. Across Canada, until 2017 physicians required a special certification from Health Canada to initiate methadone for OAT; in Halifax, no hospital-based physicians held this certification, while in Saint John, the infectious diseases physicians did [29].”

- Discussion, page 14, lines 376-384:

“Despite several years passing since our study period, the removal of Health Canada’s certification requirement to prescribe methadone for OAT, and Canada’s escalating overdose death crisis, the QEII Health Sciences Centre in Halifax still does not have addiction medicine-trained physicians or OAT providers on staff. This is typical of many hospitals in North America. Specialized addiction medicine consultation services can help close this gap, by providing expertise, increase uptake of these interventions, and assist in linking hospitalized patients to outpatient addictions treatment [25,26,32,42–44]. In response to identified gaps in care (informed by the present study), hospital-based resident physicians at the Halifax site partnered with community-based addiction physicians to provide a volunteer (unfunded) addiction medicine consultation service [27,45].

- Discussion, page 14-15, lines 352-368:

“Improving access to OAT in general medical settings requires changes in several areas, including improved education for health professionals, prioritizing access to medications on-demand, enabling multiple medication treatment options, and decreasing patients’ out-of-pocket costs [45,48]. Many patients at both hospital sites also reported injecting stimulants; managing stimulant use disorder requires other addiction treatment and harm reduction strategies beyond OAT, including psychosocial interventions and access to sterile injecting equipment [7,11].”

**Comment 2.5:** Page 13, lines 262-267: It would be helpful to provide further details on the inpatient policy enabling distribution of sterile injecting equipment. Is this a hospital-wide policy available on all wards? Are there specific designated staff who should be providing the equipment (RNs?)? Where is the physical equipment stored, and are there sufficient supplies? Do inpatients need to ask for the equipment to be provided, or is it supposed to be offered to all inpatients? Perhaps if this policy is available or documented in other publications it would be helpful to reference them. The authors should include their thoughts on why this policy may be ineffective, and what changes may be necessary.

**Authors’ response 2.5:** See response to comment #3. We have now included a copy of the policy that was active during the study period, and discussed some of its limitations including that it only included needles and syringes from the hospital formulary, while best practices suggest providing all equipment necessary to prepare and consume drugs (including cookers, filters, sterile water, etc.). The policy has been updated and is now being incorporated into health system-wide policies related to substance use and harm reduction in health care settings.:

- Methods, page 5, lines 165-166:

“The version of the relevant policy (“Intravenous Needle Exchange Policy”) that was active during the study period is presented in Fig 1.”

- Methods, page 5, lines 171-172:

**“Fig 1. Policy enabling inpatient needle and syringe distribution at Saint John Regional Hospital during the study period**.”

- Discussion, page 16, lines 449-454:

“Our study has some important limitations. First, our data came from retrospective chart review, and it is possible that hospital-based providers offered sterile injecting equipment and did not document this in the medical record. However, in Saint John the needle exchange policy at the time (Fig 1) was framed as “staff protection initiative” and so it was typical to document provision of needles to patients. At either site, if sterile injecting equipment were provided surreptitiously and not documented, this would still represent a failure to incorporate evidence-based best practices into the care plan.”

- Discussion, page 16, lines 440-445:

“The findings of the present study are now informing the development of comprehensive substance use and harm reduction policies at both hospitals. The needle exchange policy in Saint John has since been revised to reframe its focus from a “staff protection initiative” to a component of patient-centered care for people who use drugs. Best practices for hospital inpatient harm reduction policies include distribution of alcohol swabs, tourniquets, filters, cookers, sterile water, and vitamin C (which may not otherwise be on hospital formularies), in addition to needles and syringes [7,11].”

- Discussion, page 15, lines 339-401:

“We identified no documented evidence that patients with IDU-IE were offered or provided sterile injecting equipment by hospital-based health care providers at either hospital, despite a long-standing policy to facilitate this in Saint John.”

**Comment 2.6**: Limitations, page 14: The authors need to address small study size and a very specific narrow focus on patients with endocarditis, whereas there are many other inpatients without endocarditis who use injection drugs and would benefit from both sterile injecting equipment and opioid agonist therapies.

**Authors’ response 2.6:** We agree with this point and we have added it as a Limitation:

- Discussion, Limitations, page 17, lines 443-448:

“Fourth, we had a small sample size and a relatively narrow focus on patients with endocarditis, while all hospitalized patients who inject drugs could benefit from the interventions described. We selected this patient population as they represent a group at particularly high risk of death and disability from ongoing unsafe injection drug use, and should be a priority population for facilitating access to evidence-based care.”

**Comment 2.7:** Conclusion, page 14: The authors need to expand the conclusion and add future directions in advocacy or research. Do the authors suggest implementation of an inpatient addiction medicine service or increasing scope of the Infectious Diseases service to include formally treating addiction? Is further study is necessary to learn why the sterile injection equipment is unavailable, despite hospital policy? How can the unmet addiction care needs be met in the future?

**Authors’ response 2.7:** Conclusion expanded:

- Discussion, Conclusion, page 17, lines 454-461:

“To meet the needs of these patients, hospitals should employ providers with addiction medicine expertise and should develop harm reduction-oriented policies to promote patient safety. Hospital-based addiction care could be improved through integrating addiction medicine and infectious diseases specialist practice (as in Saint John Regional Hospital), and/or by establishing specialized addiction medicine consultation services and incorporating these providers into multidisciplinary endocarditis care teams. Further research is needed on the most effective ways to implement harm reduction policies in hospital to ensure resources like needle and syringe services programs are being used.”

We are grateful for your consideration of this paper and feel the suggestions have improved it significantly. We look forward to hearing from you regarding the disposition of the manuscript.

Sincerely, on behalf of all authors,

**Thomas D. Brothers, MD CISAM**

CIHR Fellow

UCL Collaborative Centre for Inclusion Health

Institute of Epidemiology and Health Care

University College London

Resident physician (General Internal Medicine & Clinician-Investigator Program)

Department of Medicine

Dalhousie University
