# Supplementary material for: Unequal access to opioid agonist treatment and sterile injecting equipment among hospitalized patients with injection drug use-associated infective endocarditis
Source: PLoS One. 2022 Jan 26;17(1):e0263156. doi: 10.1371/journal.pone.0263156 (PMC8791472; doi:10.1371/journal.pone.0263156)
Supplement: Attachment — Submitted filename: IDUIE_response_R2_220104.docx [file pone.0263156.s003.docx]

Tara Gomes, PhD
Academic Editor, *PLOS One*

22-Dec-2021

**Re: Manuscript PONE-D-21-27677R1**

Dear Dr. Gomes,

Thank you for considering our manuscript and for the opportunity to revise and re-submit. We appreciate your close reading of the manuscript and your specific questions, and we feel they have improved the quality of the manuscript. We have attempted to address your one remaining question below. We have itemized and underlined the changes (line numbers refer to those in the “tracked changes” version of the manuscript, showing “All markup”).

**Academic Editor comments:**

**Comment 1:** There is just one outstanding question that I'm hoping you can clarify. In the revised manuscript, in response to questions about recording of provision of sterile supplies in charts, the authors included the statement "However, in Saint John the needle exchange policy at the time (Fig 1) was framed as “staff protection initiative” and so it was typical to document provision of needles to patients". Is it accurate to state that it is "typical" to document provision of needles if there was no such documentation recorded in the hospital in this analysis (as per the findings of this study)? Or is this referring to documentation of provision outside of the study population in this analysis? If the authors could clarify this point, that would be helpful.

**Authors’ response**: Thank you very much for the invitation to more deeply explore and report on the practical implementation of the needle exchange policy during the study period. While the policy has never been evaluated before, when we began the study we felt we had a good understanding of the policy, as one of the original developers is a co-author (DW). In response to peer review comments, we have learned important new information that we describe below and report in the revised manuscript.

In response to a question in the first round of peer review, we had asked one of the internal medicine nurse managers in Saint John about requirements for documentation during the study period. She said that nurses and other health professionals were expected to document when needles and syringes were offered and when they were provided to hospitalized patients.

In response to your question now, we asked the nurse manager what exactly would be documented and where this documentation would occur. She said documentation would occur in either the multidisciplinary progress notes (which we had reviewed and, as we reported, found no documentation of staff providing sterile injecting equipment) or in “electronic nursing communication notes” (which we had not reviewed). We therefore reviewed these electronic nursing communication notes and found important new information.

Specifically, the electronic communication notes showed that four patients with IDU-IE in Saint John were in fact offered sterile injecting equipment, and it was provided to one of these patients. We also found that two patients in Saint John had injecting equipment confiscated, which was also only documented in these electronic nursing communication notes. We expected that patients on the internal medicine ward would be more likely to be offered sterile injecting equipment, as the policy was developed and implemented on that ward and inconsistently applied in the rest of the hospital. In fact, only 1 out of 4 patients on the internal medicine ward was offered the sterile injecting equipment (and this patient was also provided equipment) and patients on family medicine and cardiology wards were also offered equipment and made aware of the policy. Interestingly, one patient on the cardiology ward was offered equipment and another patient on the same cardiology ward had equipment confiscated. This may speak to the complexities of trying to implement harm reduction policies in a hospital setting.

We appreciate that it is unusual to discover new data after multiple rounds of peer review, and we are grateful to the academic editor for encouraging us to more deeply engage with the policy (which led to the discovery of the practice of documentation in electronic nursing notes).

Here is how we now communicate this new information in the revised manuscript:

- Abstract, Results, lines 50-53:

“In Halifax, no patients were offered sterile injecting equipment and during five hospitalizations staff confiscated patients own equipment. In Saint John, four patients were offered (and one was provided) injecting equipment in hospital, and during two hospitalizations staff confiscated patients own equipment.”

- Materials and Methods, Setting and data sources, lines 97-99:

“In both hospitals, patients with endocarditis may initially be admitted to internal medicine or to cardiology wards, and these patients might be transferred between the two services or to family medicine wards during a prolonged hospital stay.”

- Materials and Methods, Setting and data sources, lines 109-115:

“One investigator at each site (TDB and KM) performed structured chart review, extracting information on demographic and clinical factors, substance use and treatment history. This information was extracted from multidisciplinary team progress notes, nursing communication notes, specialist consultation records, physician orders, medication administration records, and hospital discharge summaries. All documentation in Halifax was available as paper charts scanned to an online system, and in Saint John there was a combination of physical paper charts and electronic progress and nursing communication notes.”

- Materials and Methods, Setting and data sources, lines 129-136:

“An inpatient program to distribute sterile injecting equipment on the internal medicine ward was established in 2007. While this policy has never been formally evaluated, based on anecdotal reports we expected it to be applied reliably for internal medicine inpatients and applied inconsistently on other wards in the hospital. The version of the relevant policy (“Intravenous Needle Exchange Policy”) that was active during the study period is presented in Fig 1. Health professionals (primarily nurses) who offered sterile injecting equipment to inpatients in Saint John were expected to document this in either multidisciplinary progress notes or in electronic nursing communication notes.”

- Results, lines 262-281:

“In Halifax, no patients had a documented offer or provision of sterile injecting equipment by hospital-based healthcare providers, but during five hospitalizations drug injecting equipment was found in patients’ rooms and confiscated by staff. In Saint John, there was documentation during four hospitalizations (27%, out of 15 hospitalizations where the patient survived) that the patient was educated about the inpatient needle and syringe distribution policy, and during one of these hospitalizations (7%) that the patient was provided needles and syringes while in hospital. There were two documented incidents of injecting equipment confiscation described by staff in Saint John. Of Saint John patients with IDU-IE admitted to the internal medicine ward (where the inpatient needle and syringe distribution policy was first developed), one out of four patients was offered equipment and no patients had equipment confiscated. The other three Saint John patients who offered equipment were on cardiology and family medicine wards. Of note, there was documentation from the same cardiology ward of patients being offered sterile injecting equipment and having equipment confiscated, at different times.”

- Discussion, lines 312-315:

“We also found that four patients were offered sterile drug injecting equipment (and one was provided equipment) at the hospital in Saint John, while no patients were offered this in Halifax. Patients at both hospitals had their own injecting equipment confiscated, despite a policy enabling needle and syringe distribution in Saint John.

- Discussion, lines 385-398:

“Patients also had differential access to sterile injecting equipment in hospital, and the equipment distribution policy in Saint John was inconsistently implemented. We identified no documented evidence that patients with IDU-IE were offered or provided sterile injecting equipment by hospital-based health care providers in Halifax. In Saint John, where there has been a hospital policy facilitating needle and syringe distribution to hospital inpatients since 2007, patients were offered sterile injecting equipment during four out of 17 hospitalizations. We did not capture information on the reasons that not all patients were offered equipment, patients may not be made aware of the policy or may feel unsafe disclosing ongoing substance use [38,51,52]. Some patients may have stated goals to abstain from injecting, especially as most patients were either already on OAT or initiated OAT in hospital. Many of the patients with IDU-IE were acutely ill during much of their hospital stay, and so the health care team may not have considered the possibility of ongoing substance use in hospital or the potential benefits of offering sterile injecting equipment. Overall, the inconsistent implementation suggests that a written policy is not enough to ensure reliable implementation, which has also been observed at one other Canadian hospital with an inpatient policy to distribute sterile injecting equipment [50].

- Discussion, lines 400-424:

“Several patients at both hospitals were found to have injecting equipment in their hospital rooms and these were confiscated, despite the policy enabling needle and syringe distribution in Saint John and that no hospital policy exists banning sterile injecting equipment in hospital in Halifax. One ward in Saint John offered sterile injecting equipment to one patient with IDU-IE and confiscated it from another. Further, some of this confiscated equipment likely came from the publicly funded needle and syringe distribution programs in both cities; Halifax’s program is located just a few blocks away from the hospital.

- Conclusion, lines 481-483:

“Further research is needed on the most effective ways to implement harm reduction policies in hospital to ensure resources like needle and syringe services programs are being offered and provided reliably.”

Within these newly reviewed electronic nursing communications notes, we also identified documentation of concerns regarding illicit or non-medical substance use in hospital for one additional patient, and updated the Abstract and Table 2 with this information:

- Abstract, lines 53-56:

“Concerns regarding undertreated pain or opioid withdrawal were documented during 66% (25/38) of hospitalizations, and in-hospital illicit or non-medical drug use during 31% (12/38).”

- Results, lines 277-279:

“Illicit or non-medical substance use was documented in 31% (12/38) of all hospitalizations, and patient-initiated discharges against medical advice occurred in 11% (4/38) of all hospitalizations.”

- Table 2 (Saint John column):

“Illicit or non-medical substance use in hospital: 5 (29%)”

We also identified and fixed an important typo, which is that the 21 hospitalizations in Halifax and 17 hospitalizations in Saint John add up to 38 total – rather than 39 total – hospitalizations.

We are grateful for your consideration of this paper and feel the suggestions have improved it significantly. We look forward to hearing from you regarding the disposition of the manuscript.

Sincerely, on behalf of all authors,

**Thomas D. Brothers, MD CISAM**

CIHR Fellow

UCL Collaborative Centre for Inclusion Health

Institute of Epidemiology and Health Care

University College London

Resident physician (General Internal Medicine & Clinician-Investigator Program)

Department of Medicine

Dalhousie University
