# Supplementary material for: Unequal access to opioid agonist treatment and sterile injecting equipment among hospitalized patients with injection drug use-associated infective endocarditis
Source: PLoS One. 2022 Jan 26;17(1):e0263156. doi: 10.1371/journal.pone.0263156 (PMC8791472; doi:10.1371/journal.pone.0263156)
Supplement: Attachment — Submitted filename: IDUIE_response_R3_220107.docx [file pone.0263156.s004.docx]

Tara Gomes, PhD
Academic Editor, *PLOS One*

7-Jan-2022

**Re: Manuscript PONE-D-21-27677R3**

Dear Dr. Gomes,

Thank you for considering our manuscript and for the opportunity to revise and re-submit. We appreciate your close reading of the manuscript and your specific questions, and we feel they have improved the quality of the manuscript. We have attempted to address your two remaining questions below. We have itemized and underlined the changes (line numbers refer to those in the “tracked changes” version of the manuscript, showing “All markup”).

**Academic Editor comments:**

**Comment 1:** You mention that "*Of note, there was documentation from the same cardiology ward of patients being offered sterile injecting equipment and having equipment confiscated, at different times*". It isn't clear from this statement whether the confiscation occurred in the same patient who was offered the sterile equipment, or in different patients. Can you revise to clarify this point?

**Authors’ response 1**: Thank you for catching this. These were different patients on the same ward. We have edited the text to clarify:

- Results, lines 243-244:

“Within the same cardiology ward, one patient was offered sterile injecting equipment and a different patient had equipment confiscated.”

**Comment 2:** The finding of a high proportion of IE cases using hydromorphone is one that I hadn't initially noticed, and could garner some attention given the debate around hydromorphone as safer supply. From your sentence "*primarily with hydromorphone tablets and none with heroin, which is consistent with the illicit/criminalized drug supply available in the local communities*" and the accompanying citation, it seems clear that this finding is NOT due to safer supply, but due to the illicit drug supply in Nova Scotia at the time of study. Has the illicit drug supply has changed in Nova Scotia since 2017 (similar to other jurisdictions in Canada where fentanyl is now predominantly driving overdose rates)? To avoid misinterpretation of these findings (or inappropriate assignment of the hydromorphone IE risks to safer supply), I would suggest reiterating in the sentence highlighted above that this finding is consistent with the illicit drug supply in Nova Scotia *at the time of this study.* You may also want to expand this to indicate whether you believe this finding is suggestive of IE among people accessing hydromorphone for safer supply (I assume not - I don't believe that safer supply with HM would have been prevalent during your study period) to help avoid misinterpretation or misapplication of these findings to the safer supply debate.

**Authors’ response 2:** We agree it is important to help readers understand the context for the high rate of hydromorphone use in our sample and that this case-only study design cannot speak to risk factors for IE. To make sure these data do not get misapplied in the “safe supply” debate around hydromorphone prescribing and infection risk, we have moved the consideration of reported hydromorphone use to its own paragraph and added this discussion:

- Discussion, lines 381-403:

“Among patients with IDU-IE in both Halifax and Saint John sites who reported injection opioid use, all patients used diverted or non-prescribed pharmaceutical opioids (primarily hydromorphone tablets) and none reported heroin or fentanyl use. This pattern is consistent with the drug supply available in these communities during the study period, as nearly all illicit or non-prescribed opioids were pharmaceutical and there has been very little availability of heroin for several decades [33,61,62,62]. Illicitly-manufactured fentanyl and its analogues have become more available since the study period, but pharmaceutical opioids still comprise the majority of illicit or non-prescribed opioids in these communities [63]. As all patients in our study had IDU-IE, we cannot say from these data whether injecting pharmaceutical opioid tablets increases risk for serious bacterial infections more than injecting heroin or fentanyl. While one study identified an association between controlled-release hydromorphone prescriptions and IDU-IE in Ontario [64], a small minority of patients with IDU-IE in Ontario have recent prescriptions for hydromorphone [6,64]. Increasing rates of serious injection drug use-associated bacterial infections have been observed in many jurisdictions [4,5], and in Ontario appear to parallel transitions in the drug supply towards increasing use of illicitly-manufactured fentanyl [6]. Known risk factors for serious injecting-related infections include more frequent injecting (which has been associated with fentanyl and with stimulants), re-use of contaminated needles and filters, and subcutaneous injecting [65]. Social determinants, including homelessness (impacting sterile drug preparation) and criminalization and policing practices (leading to rushed, subcutaneous injections), also influence risk [5]. We did not extract information on patients’ current or recent outpatient medication prescriptions to determine whether patients had recent prescriptions for these opioids, and we did not have reliable information on housing status. Purposefully prescribing pharmaceutical opioids for injection as an alternative to the unregulated street drug supply, known as “safe supply” prescribing [66,67], was not known to be a practice in these communities during the study period [62].

We are grateful for your consideration of this paper and feel the suggestions have improved it significantly. We look forward to hearing from you regarding the disposition of the manuscript.

Sincerely, on behalf of all authors,

**Thomas D. Brothers, MD CISAM**

CIHR Fellow

UCL Collaborative Centre for Inclusion Health

Institute of Epidemiology and Health Care

University College London

Resident physician (General Internal Medicine & Clinician-Investigator Program)

Department of Medicine

Dalhousie University
